# Increased adherence to treatment guidelines in patients with urinary tract infection in primary care: A retrospective study

**DOI:** 10.1371/journal.pone.0214572

**Published:** 2019-03-28

**Authors:** Helena Kornfält Isberg, Katarina Hedin, Eva Melander, Sigvard Mölstad, Anders Beckman

**Affiliations:** 1 Department of Clinical Sciences, Malmö, Family Medicine, Lund University, Malmö, Sweden; 2 Futurum, Region Jönköping County, Jönköping, Sweden; 3 Department of Medical and Health Sciences, Linköping University, Linköping, Sweden; 4 Regional Centre for Communicable Disease Control, Malmö, Sweden; 5 Department of Translational Medicine, Lund University, Malmö, Sweden; Institut Hospital del Mar d’Investigacions Mediques, SPAIN

## Abstract

**Background:**

Urinary tract infection (UTI) is common in primary care and leads to a high number of antibiotic prescriptions. Antimicrobial resistance is a global health problem; better antimicrobial prescribing is one way to limit antimicrobial resistance. We aimed to describe the number of consultations for patients diagnosed with lower urinary tract infection (LUTI) and pyelonephritis and changes in prescribing of antibiotics to men and women with LUTI and pyelonephritis in Swedish PHC between the years 2008 and 2013.

**Methods:**

We performed a descriptive study of changes in UTI diagnosis and antibiotic prescribing in UTI for the years 2008, 2010 and 2013. The Primary Care Record of Infections in Sweden, a database regarding diagnosis linked antibiotic prescribing in primary care, was analyzed concerning data for men and women of all ages regarding UTI visits and antibiotic prescribing. The results were analyzed in relation to current national guidelines.

**Results:**

There was a variability in consultation incidence for LUTI with an increase between 2008 and 2010 and a decrease between 2010 and 2013, resulting in a slight rise in consultation incidence between 2008 and 2013. The use of recommended nitrofurantoin or pivmecillinam in LUTI in women increased from 54% in 2008 to 69% in 2013. Fluoroquinolones or trimethoprim were prescribed in 24% of LUTI cases in women in 2008 and in 7% of cases in 2013. Prescriptions of pivmecillinam or nitrofurantoin in male LUTI cases increased from 13% in 2008 to 31% in 2013. Fluoroquinolones or trimethoprim were prescribed in 54% of male LUTI cases in 2008 and 32% in 2013.

**Conclusions:**

Swedish GPs seem to follow national guidelines in the treatment of LUTI in women. In male LUTI cases, the prescriptions of fluoroquinolones remain high and further research is needed to follow prescription patterns and enhance more prudent prescribing to this group of patients.

## Introduction

The increasing frequency of bacteria resistant to antibiotics is a global health problem. Inappropriate use of antibiotics favors the emergence and selection of resistant bacterial strains [1]. In general, the proportions of bacterial resistance to clinically important antimicrobials are low in Sweden. However, increasing resistance levels to trimethoprim and ciprofloxacin were observed with *Escherichia coli* and this was one of the reasons behind the revised recommendations for the treatment of lower urinary tract infection (LUTI) in women, which were published in 2007 and for men in 2014 [2, 3]. Pivmecillinam and nitrofurantoin are now recommended as first choice antibiotics when treating uncomplicated LUTI in both women and men. Nitrofurantoin is recommended also to elderly patients but should not be used in patients with reduced kidney function (glomerular filtration rate < 40 ml/min). Since 2007 (women) and 2014 (men), the use of cephalosporins or fluoroquinolones is no longer recommended in Sweden when treating LUTI [2–4]. For pyelonephritis the recommended drugs are still fluoroquinolones, trimethoprim-sulfamethoxazole and cephalosporins. In Swedish guidelines, patients with structural or functional alterations in the urinary tract are defined as complicated UTI and special considerations should be taken before treatment with antibiotics. Other antibiotics than the recommended first choice antibiotic could be prescribed. Based on sales data of UTI antibiotics, sales of fluoroquinolones and trimethoprim have decreased and sales of recommended antibiotics have increased following the new recommendations [5]. The total prescribing rate of UTI antibiotics to women aged 65 years and older remains high [5].

In Swedish primary health care (PHC), LUTI is the greatest cause of antibiotic prescribing [6]. Among women with suspected LUTI, 85% were prescribed antibiotics [6] and 10% of all women older than 18 years of age were annually prescribed antibiotics once or more due to LUTI [4]. LUTI in men is not as common as in women and there are only a few studies concerning LUTI in men in primary care. In one study the mean age for men consulting with symptoms suggestive for LUTI was 61 years and the incidence increased with age [7].

Acute pyelonephritis is far less common than LUTI. The incidence of pyelonephritis is highest among young women, followed by infants and elderly people. The annual rates of outpatient pyelonephritis were 12–13 cases in women and 2–3 cases in men per 10,000 respectively [8].

Using large diagnosis linked prescribing registers, trends in antibiotic prescribing in primary care in the UK during 1995–2011 and in the Netherlands during 2007–10 have been described, [9, 10]. However, antibiotic recommendations and resistance rates differ between the countries and the studies were designed to describe antibiotic prescribing in general, not in UTI exclusively. Previous Swedish studies on UTI and antibiotic prescribing have been performed during the course of one week [4, 11] or in a single part of Sweden during one year [12]. Data covering many patients is needed in order to describe trends in prescribing and adherence to antibiotic guidelines diagnosis linked prescribing. To improve antibiotic stewardship, we need to be able to follow antibiotic prescribing and the clinical diagnosis connected to the prescription.

We aimed to describe the number of consultations for patients diagnosed with LUTI and pyelonephritis and changes in prescribing of antibiotics to men and women with LUTI and pyelonephritis in Swedish PHC between the years 2008 and 2013.

## Material and method

This retrospective descriptive study was based on information from the PRIS record for the years 2008, 2010 and 2013.

### PRIS

To evaluate the prescribing rate and the adherence to clinical guidelines, the PRIS record (Primary Care Record of Infections in Sweden) was created by the Unit of Research and development (R&D) in Primary Care in Jönköping in 2007. The PRIS record consists of diagnosis based data and uses information from medical records from patients in primary health care.[6, 13] The PRIS record was closed in 2014.

Primary Health Care Centers (PHCCs) from all parts of Sweden were invited each year to report patient data on all infectious diagnoses to the PRIS record. In Sweden, general practitioners (GP) work in primary health care and a full time GP handles 1500–2000 registered persons. The inhabitants are encouraged to register with any public or private PHCC within their region. In 2008 a total of 47 PHCCs with 460,529 registered persons were included in the PRIS register. An increasing number of PHCCs subsequently joined the PRIS register and in 2013 a total of 88 PHCCs with 785, 070 registered persons were included. Included PHCC were both small and large units, private and public, situated in rural and urban areas in all parts of Sweden. The number of registered persons in the PRIS record in 2013 represented 8% of Sweden’s population. The ages for the patients included in the study was known but the age distribution of all registered persons was not known. Due to the large number of registered persons in the material, the age- distribution of total registered persons for all included PHCC was assumed to correspond to the age-distribution of the total inhabitants of Sweden as of December 31^st^ for the respective year.

All visits during office hours that resulted in a diagnosis of infection were registered according to the International Classification of Disease and Related Health Problems-Tenth Revision (ICD-10) introduced by the World Health Organization (WHO). Prescribed antibiotics were registered according to the Anatomical Therapeutic Classification System (ATC). The ATC-coding system is used to classify drugs in order to facilitate studies on drug-use in Sweden [13]. Further data collected in the PRIS record included patient identification number (encrypted ID number), date of visit, patient age and sex and, if applicable, laboratory testing. Some patient visits had more than one registered diagnosis; the diagnoses in PRIS have therefore been ranked. The highest ranked is the diagnosis most likely to cause an antibiotic prescription. Of the most common UTI diagnoses, pyelonephritis has the highest rank, followed by acute cystitis and UTI that have the same ranking. If a contact has generated more than one diagnosis, only the one that has the highest ranking has been retained. The same ranking system was used all years [14].

### Data processing

PRIS-data from the years 2008, 2010 and 2013 were analyzed focusing on visits related to UTI. Inclusion criteria were a diagnosis classified as UTI (S1 Appendix). For each year all registered contacts with a diagnosis classified as UTI were included. Variables retrieved from the register for each investigated year were diagnosis, sex, age (all ages) and prescribed antibiotic. To calculate the number of diagnoses per 1000 registered person and year, the number of diagnoses was used as the numerator and the mean number of registered persons per PHCC for 2008 (460529), 2010 (556192) and 2013 (785070) as the denominator. The calculation of the number of antibiotic prescriptions per 1000 registered person and year was done in the same way but the number of antibiotic prescriptions was used as the numerator. Gender specific incidence was calculated by dividing the number of incident LUTI and pyelonephritis by the total number of registered women and men at all included PHCC for each year. The same procedure was used to calculate the registered number of prescriptions of antibiotics and type of antibiotic prescribed. Each PHCC provided data on the number of registered persons at their clinic, calculated as a mean, per year, thus the calculations in the study are based on the mean number of registered persons at the included PHCC for each investigated year.

Included patients were stratified into age-groups. The incidence of UTI and prescribed antibiotics was described by age group 0–15, 16–30, 31–50, 51–70, 71- years. The same age-groups were used in both men and women. Intervals in age-groups were not equal in size but were defined according to the following: children, young adults, adults, older alduts and elderly. Calculations on incidence of UTI per 1000 registered person and year for men and women in each age group were based on figures on age distribution in Sweden from Statistics Sweden for the actual years [15, 16].

Percentage of consultations due to infections, prescribed antibiotics and prescribing rate from the 37 PHCCs that participated all investigated years (2008, 2010 and 2013) were analyzed separately and compared to that of all included PHCCs. This sensitivity analysis was published by Tyrstrup et al [16]. Further sensitivity analyses were done in the present study where percentage of first choice UTI antibiotics, nitrofurantoin and pivmecillinam in women and proportion of treated patients were calculated. Data did not differ between the groups regarding choice and proportion of antibiotic prescriptions in UTI (S2 Appendix).

### Statistics

The data collected in the study were analyzed using Excel (Microsoft. Microsoft Excel. Redmond. Washington:Microsoft, 2010. Computer Software) and SPSS Statistics 22 (SPSS, Inc Chicago, IL). Data were mainly descriptive with numbers and frequencies presented in tables. Differences between groups were tested using the two-sided Chi-square test for categorical variables (CHISQ.TEST function of Microsoft Excel). *P*-values ≤ 0.05 were considered statistically significant. We reported 95% confidence intervals for ratios, assuming a normal distribution.

### Ethics approval

Confidentiality for patients was ensured by using a one-way encrypted ID-number. The study was approved by the Regional Ethical Review Board in Linköping, Sweden, for the PRIS register. (Dnr 2010/227-31).

## Results

The total number of registered persons and the number of PHCCs included in the PRIS database increased from 2008 to 2013 (Table 1). Consultation rates and antibiotic prescribing rates were calculated for all participating PHCCs and for the 37 PHCCs participating all three years and did not differ between the two groups. The proportion of prescriptions of first line and second line antibiotics in LUTI in women for the 37 PHCCs that participated all years did not differ from the total number of participating PHCC (S2 Appendix).

**Table 1:** Description of the study population.

Year	2008	2010	*p*-value^a^	2013	*p*-value^b^
Population (Number of registered persons)	460 529	556 192		785 070	
Women, proportion %	50	50		50	
Number of Primary Healthcare Centers (PHCC)	47	58		88	
**Consultations**					
Consultations (all causes) *n*	662 184	809 964		1 085 829	
Consultations due to infections *n*	210 388	245 344		318 976	
Consultations due to infections % (95% CI)	31.8 (31.7–31.9)	30.3 (30.2–30.4)	<0.001	29.4 (29.3–29.5)	<0.001
Consultations regarding LUTI, *n*	18312	24137		33308	
Consultations regarding LUTI % (95% CI)	8.7 (8.6–8.8)	9.8 (9.7–10.0)	<0.001	10.4 (10.3–10.5)	<0.001
Consultations due to LUTI per 1000 registered person and year (95% CI)	39.8 (39.2–40.3)	43.4 (42.9–43.9)	<0.001	42.4 (42.0–42.9)	<0.001
Consultations due to LUTI per 1000 women and year (95% CI)	70.3 (69.3–71.4)	76.1 (75.1–77.1)	<0.001	73.0 (72.2–73.8)	<0.001
Consultations due to LUTI per 1000 registered men and year (95% CI)	9.2 (8.8–9.6)	10.6 (10.2–11.0)	<0.001	11.9 (11.5–12.2)	<0.001
Consultations due to pyelonephritis per 1000 registered person and year (95% CI)	1.3 (1.2–1.4)	1.5 (1.4–1.6)	0.001	1.3 (1.2–1.4)	0.002
Consultations due to pyelonephritis per 1000 registered women and year (95% CI)	1.9 (1.7–2.1)	2.3 (2.1–2.4)	0.005	1.9 (1.8–2.0)	<0.001
Consultations due to pyelonephritis per 1000 registered men and year (95% CI)	0.7 (0.6–0.8)	0.8 (0.7–0.9)	0.130	0.8 (0.7–0.9)	0.676

*p*-values calculated using chi-2 test

^a^Comparison between year 2008 and 2010

^b^Comparison between year 2010 and 2013

### Lower urinary tract infection (LUTI)

#### Women

**Fig 1:**
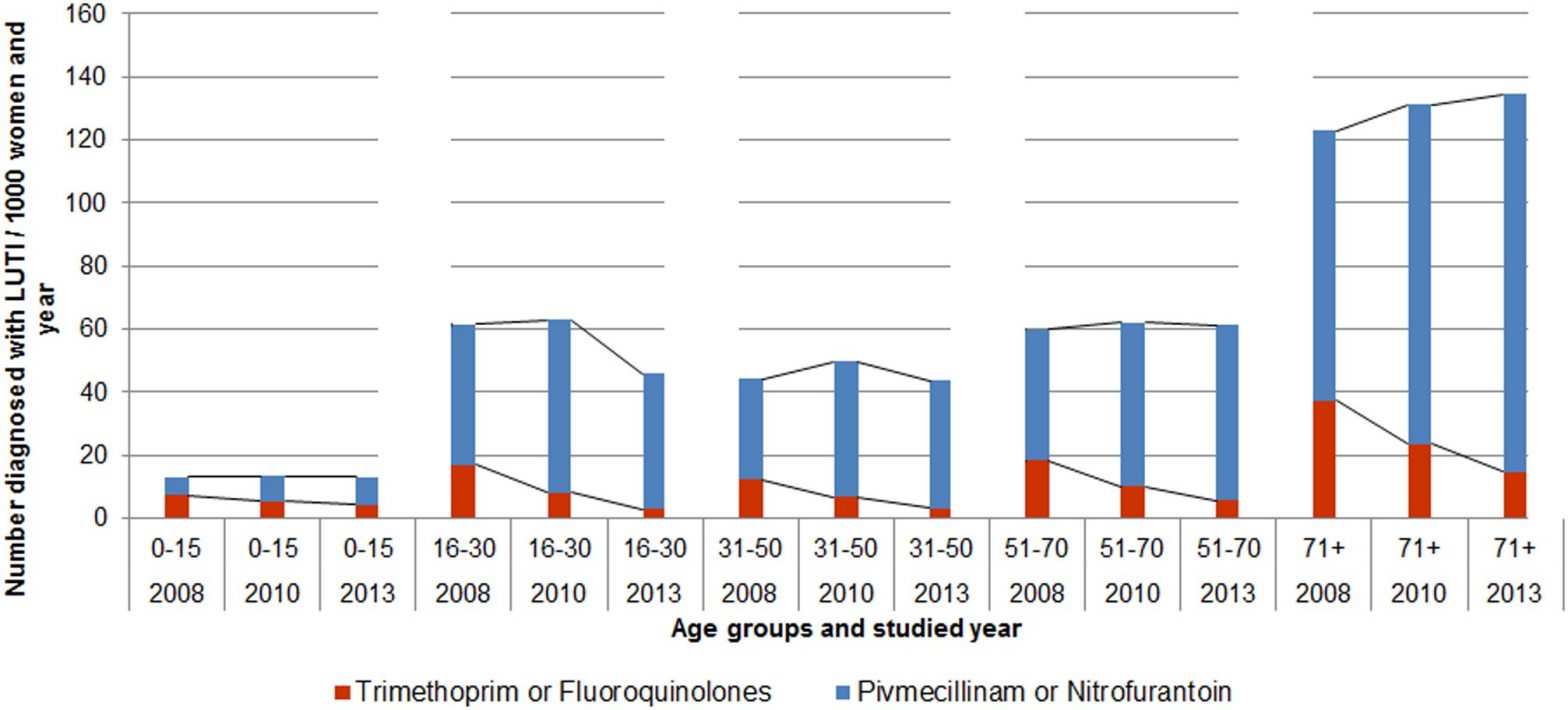
Antibiotic treatment in women diagnosed with lower urinary tract infection, according to age-group in the years 2008, 2010 and 2013.

The number of antibiotic prescriptions to women with LUTI was 60/1000 registered women in 2008, 63/1000 in 2010 and 58/1000 registered women in 2013. The number of women with LUTI linked to an antibiotic prescription, who were prescribed pivmecillinam or nitrofurantoin, increased from 54% in 2008 to 67% in 2010 and to 69% in 2013. The proportion of trimethoprim or fluoroquinolones decreased from 24% in 2008 to 13% in 2010 and to 7.% in 2013 (Table 2). The number of prescriptions of UTI antibiotics per age group in the years 2008, 2010 and 2013 are presented in Fig 1. The predominant age group for LUTI was 71 years and older.

**Table 2:** PRIS data on women and men diagnosed with lower urinary tract infection. Antibiotic prescriptions to women and men with LUTI.

	Percent (95% CI)									
	Women					Men				
	2008 n = 16189	2010 n = 21173	*p*-value^a^	2013 n = 28647	*p*-value^b^	2008 n = 2123	2010 n = 2953	*p*-value^a^	2013 n = 4653	*p*-value^b^
**No antibiotic**	14.2 (13.7–14.8)	17.4 (16.9–17.9)	<0.001	20.5 (20.0–21.0)	<0.001	24.2 (22.3–26.0)	31.3 (29.6–33.0)	<0.001	32.0 (30.6–33.3)	0.529
**Pivmecillinam**	37.3 (36.5–38.0)	39.5 (38.8–40.2)	<0.001	40.7 (40.1–41.2)	0.008	8.2 (7.0–9.4)	10.5 (9.4–11.6)	0.005	17.1 (16.0–18.2)	<0.001
**Nitrofurantoin**	17.1(16.5–17.7)	27.4 (26.8–28.0)	<0.001	28.7 (28.2–29.2)	<0.001	5.2 (4.2–6.1)	8.4 (7.4–9.4)	<0.001	13.5 (12.5–14.4)	<0.001
**Trimethoprim**	18.0 (17.4–18.5)	9.4 (9.0–9.8)	<0.001	4.8 (4.6–5.1)	<0.001	14.1 (12.6–15.6)	10.6 (9.5–11.7)	<0.001	5.9 (5.3–6.6)	<0.001
**Fluoroquinolones**	6.1 (5.7–6.5)	3.2 (3.0–3.5)	<0.001	2.5 (2.3–2.6)	<0.001	40.3 (38.2–42.4)	34.2 (32.5–35.9)	<0.001	26.5 (25.3–27.8)	<0.001
**Other**^c^	7.4 (7.0–7.8)	3.1 (2.9–3.3)	<0.001	2.8 (2.6–3.0)	0.055	8.1 (6.9–9.2)	4.9 (4.2–5.7)	<0.001	5.0 (4.4–5.7)	0.868

*p-*values were calculated using the Chi-square test, CI = confidence interval

^a^Comparison between year 2008 and 2010

^b^Comparison between year 2010 and 2013

^c^ Other: Tetracyclines, Penicillins with extended spectrum (Amoxicillin), Beta-lactamase sensitive penicillins, Cephalosporins (1^st^ and 2^nd^ generation), Carbapenems, Macrolides, Lincosamides (Clindamycin), sulfamethoxazole-trimethoprim

#### Men

The number of antibiotic prescriptions to men with LUTI was 7/1000 in 2008, 7/1000 in 2010 and 8/1000 men in 2013.

**Fig 2:**
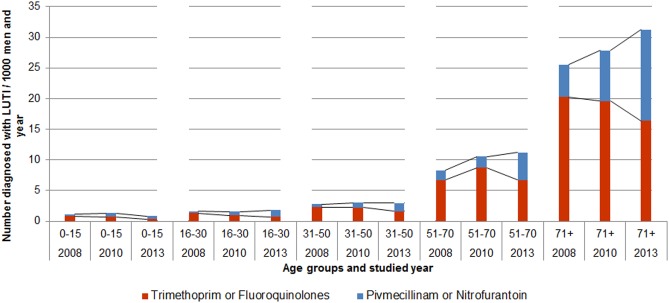
Antibiotic treatment in men diagnosed with lower urinary tract infection, according to age-group in the years 2008, 2010 and 2013.

Antibiotic prescriptions to men diagnosed with LUTI are presented in Table 2. The number of prescriptions of UTI antibiotics per age-group in the years 2008, 2010 and 2013 are presented in Fig 2.

LUTI was not common among men younger than 50 years of age. The frequency of prescriptions for LUTI in men increased with age (Fig 2). The proportion of men with LUTI linked to an antibiotic prescription, who were prescribed pivmecillinam or nitrofurantoin, increased from 13% in 2008 to 19% in 2010 and to 31% in 2013. The number of trimethoprim or fluoroquinolones decreased from 54% in 2008 to 45% in 2010 and to 32% in 2013 (Table 2).

### Pyelonephritis

#### Women

Women made up almost three-quarters of visits by patients diagnosed with pyelonephritis. Pyelonephritis was most common among women aged 16–30 years (1.07, 1.30, 1.03 per 1000 registered persons and year in 2008, 2010 and 2013) and among women 51 years and older (1.01, 1.27, 1.04 per 1000 registered persons and year in 2008, 2010 and 2013). Fluoroquinolones were the most common antibiotics prescribed (Table 3).

**Table 3:** PRIS data on women and men diagnosed with pyelonephritis. Antibiotic prescriptions to women and men with pyelonephritis.

	Percent (95% CI)									
	Women					Men				
	2008 n = 437	2010 n = 633	*p*-value^a^	2013 n = 741	*p*-value^b^	2008 n = 157	2010 n = 223	*p*-value^a^	2013 n = 302	*p*-value^b^
**No antibiotic**	38.4 (33.9–43.0)	43.6 (39.7–47.5)	0.092	38.6 (35.1–42.1)	0.060	46.5 (38.7–54.3)	43.9 (37.4–50.5)	0.623	35.8 (30.4–41.2)	0.058
**Fluoroquinolones**	48.5 (43.8–53.2)	42.5 (38.6–46.3)	0.052	49.1 (45.5–52.7)	0.014	47.8 (40.0–55.6)	48.9 (42.3–55.4)	0.831	55.3 (49.7–60.9)	0.145
**Sulfamethoxazole-trimethoprim**	5.0 (3.0–7.1)	3.9 (2.4–5.5)	0.395	5.3 (3.7–6.9)	0.249	2.5 (0.1–5.0)	0.9 (-0.3–2.1)	0.204	4.3 (2.0–6.6)	0.021
**Ceftibuten**	2.5 (1.0–4.0)	2.2 (1.1–3.4)	0.745	4.2 (2.7–5.6)	0.041	0 (0–0)	0.4 (-0.4–1.3)	0.401	2.0 (0.4–3.6)	0.129
**Other**^c^	5.5 (3.4–7.6)	7.7 (5.7–9.8)	0.152	2.8 (1.6–4.0)	<0.001	3.2 (0.4–5.9)	5.8 (2.8–8.9)	0.232	2.6 (0.8–4.5)	0.066

*p-*values were calculated using the Chi-square test, CI = confidence interval

^a^Comparison between year 2008 and 2010

^b^Comparison between year 2010 and 2013

^c^Other: Pivmecillinam, nitrofurantoin, cefadroxil, amoxicillin, trimethoprim, clindamycin, phenoxymethylpenicillin

#### Men

Visits related to pyelonephritis were uncommon in younger men (16–30 years 0.07, 0.09 and 0.07 per 1000 registered persons and year in 2008, 2010 and 2013). The incidence rates increased with age and pyelonephritis was most common in the age-group 71 years and older (0.82, 1.12, 1.20 per 1000 registered persons and year in 2008, 2010 and 2013). Fluoroquinolones were the most commonly prescribed antibiotics (Table 3).

### Antibiotic prescribing to patients without UTI diagnosis

The frequency of prescriptions of antibiotics without a UTI diagnosis in the PRIS database decreased from 21% in 2008 to 15% in 2010 and 14% in 2013. Antibiotic prescriptions with no registered diagnosis could due to prescription of pivmecillinam and nitrofurantoin (that are prescribed to treat UTI exclusively) be allocated to probable UTIs in 31% (2008), 25% (2010) and 36% (2013) of the cases.

## Discussion

This register based study on LUTI showed a variability in consultation incidence with an increase between 2008 and 2010 and a decrease between 2010 and 2013, resulting in a slight rise in consultation incidence due to LUTI between 2008 and 2013. The proportion of patients not receiving antibiotic prescription in spite of a diagnosis of LUTI increased, especially in women. In both women and men the number of prescriptions of pivmecillinam and nitrofurantoin increased and the prescriptions of trimethoprim and fluoroquinolones decreased.

### Findings in relation to current guidelines

According to the Swedish treatment recommendations from 2007 the first choice antibiotics for the treatment of LUTI in women were pivmecillinam and nitrofurantoin. Trimethoprim could be used as a second choice antibiotic but only after urinary culture. Prescribers were also recommended to minimize the use of fluoroquinolones [2]. According to Swedres/Svarm data based on all prescriptions from outpatient care, the use of the two first-line drugs (pivmecillinam and nitrofurantoin) has increased and the use of trimethoprim and fluoroquinolones has decreased from 2008–2013, which is in accordance with recommendations and in-line with PRIS data [5, 17]. PRIS data also shows that numbers of prescriptions of second choice antibiotics are low which indicates good adherence to guidelines.

Expert recommendations regarding male LUTI were formally released in 2014. Nitrofurantoin and pivmecillinam are now recommended as first choice antibiotics also in the treatment of LUTI without fever in men [3, 5, 17].

The Swedish strategic programme against antibiotic resistance (STRAMA) recommended that fluoroquinolones should make up less than 10% of UTI antibiotics in women aged 18–79 years [18]. Sales data, based on all prescriptions from outpatient care, also including patients from hospital clinics, showed that the levels of prescriptions of fluoroquinolones to women were 13% in 2013 and 13.8% in 2014; no Swedish region did reach the STRAMA goal for prescription of fluoroquinolones [19]. The proportion of prescribed fluoroquinolones to women in our study, only including diagnosis linked prescriptions in primary healthcare and no visits during after-office hours, is much lower than expected based on sales data; 3% of women with LUTI were prescribed fluoroquinolones according to PRIS-data. However, sales data does not include a diagnosis and fluoroquinolones are used for a number of other diagnoses. Fluoroquinolones and Sulfamethoxazole- trimethoprim (after culture) are recommended first choice antibiotics in cases of pyelonephritis in both men and women. However, pyelonephritis represents a small proportion of infections diagnosed in primary health care and contributes to a low extent regarding the total share of prescriptions of fluoroquinolones Changes in the pattern of prescribing in favor of narrower spectrum antibiotics in male LUTI were seen already in 2010. This is most likely due to ongoing discussions concerning antimicrobial resistance and the possibility to treat LUTI in men with narrower spectrum antibiotics but could also be interpreted as poor adherence to guidelines. Local recommendations preceding national recommendations could also have affected the prescription pattern in male LUTI.

The increased ratio of narrow spectrum antibiotics and a decrease in fluoroquinolones in the treatment of LUTI seen in the present study is positive and one step in the work to decrease the use of fluoroquinolones, which is of importance, since they often promote the development of antimicrobial resistance. We still need to follow prescription patterns in this group of patients and we also have to evaluate the effect of narrower antibiotics in the treatment of LUTI, especially in men where few studies have been done.

### Treatment recommendations in different countries

The bacterial etiology, resistance patterns and national recommendations for antibiotic therapy in LUTI differ between European countries. Treatment recommendations are most likely based on traditions, availability of antibiotics and differences in antimicrobial resistance patterns. In a 2013 article, national recommendations from six European countries were described. Nitrofurantoin and pivmecillinam were the most agreed upon first choice antibiotic treatments for women and were recommended in five and three countries respectively [20].

According to the present study, Swedish GPs show good adherence to clinical recommendations in the treatment of female LUTI. In the UK, diagnosis linked prescribing data were used to describe trends in prescribing of UTI antibiotics in primary care during 1995–2011. The use of recommended short course trimethoprim for UTI in women increased from 8% to 50% between 1995 and 2011 [9]. More recent data from the UK shows that it was still 50% during 2013–2015 [21].

To implement new recommendations in health services, a wide variety of conditions must be satisfied [22, 23]. Research on implementation in healthcare suggests that the context, evidence, evaluation and feedback are important factors to be considered when implementing new healthcare strategies [24]. To achieve goals for implementation, individual changes and changes on a group and organizational level are needed [24]. Efforts to implement guidelines and to reduce antibiotic use in patients with UTI are ongoing and the Swedish strategic programme against antibiotic resistance (Strama) is constantly working closely with prescribers at the local level to monitor antibiotic use and to reduce unnecessary prescribing [25, 26].

### Strengths and limitations

The main strengths of the present study are the large number of patients studied and the inclusion of male LUTI. In order to monitor the use of antibiotics in the public health care system, we need register-based diagnosis linked prescribing data. The PRIS register, which has more than half a million infection-related visits, gives us the opportunity to follow changes in antibiotic prescribing and adherence to clinical guidelines over time. Previous studies conducted in Sweden on prescribing in PHC are mainly based on sales data in order to assess whether antibiotics are used adequately; diagnosis linked prescription data from PHC is needed.

Previous studies describing trends in antimicrobial prescribing in primary care have not shown data on male LUTI [9, 10]. To our knowledge, this is the first study including such a large number of registered persons describing antibiotic treatment of male LUTI patients in primary care over time. We did not find any study describing adherence to clinical recommendations in this population.

The number of PHCCs included in PRIS increased progressively from 2008 to 2013, which may have affected measurements over that period. We did a sensitivity analysis and did not find any difference between the PHCCs that participated all the years and the total PHCCs (S2 Appendix).

One limitation of the study is the possibility that PHCCs interested in participating in the PRIS register may be more interested in quality assessment and more prone to follow clinical guidelines when treating UTI than PHCCs not participating in PRIS. We found many visits registered as UTI or pyelonephritis without prescribing of antibiotics. Considering the serious condition of pyelonephritis this could be alarming. However, we believe that many patients diagnosed with pyelonephritis were referred to the emergency ward and thus not prescribed antibiotics at the PHCC. Furthermore, this could also represent patients revisiting for check-up after the infection.

A limitation is that PRIS does not contain information about treatment failure, drug allergies, adverse events from antibiotic treatment or recurrent infections which could interfere with the number of antibiotic prescriptions and the choice of antibiotic in relation to UTI diagnosis.

One limitation with register-based studies is that we are dependent on clinicians registering the correct diagnosis when prescribing antibiotics. The clinicians are not obliged to register a diagnosis when prescribing antibiotics and in some cases clinicians may think that antibiotics with only one indication (i.e.nitrofurantoin, pivmecillinam) can replace the need for a diagnostic code. A diagnosis labelling may legitimate an antibiotic prescription in cases where the clinical UTI diagnosis is doubtful. This study found that a diagnosis was often missing when prescribing nitrofurantoin and pivmecillinam, we therefore believe that the incidence of UTI in PHC is higher than described in this study.

## Conclusion

The major change in prescribing of antibiotics commonly used in the treatment of LUTI among patients in primary health care is in line with the Swedish guidelines published in 2007 for women and in 2014 for men. Swedish GPs follow clinical recommendations in the treatment of LUTI in women. Prescriptions of nitrofurantoin and pivmecillinam in this group increased during the studied period and prescriptions of fluoroquinolones and trimethoprim decreased. In male LUTI patients, the prescriptions of fluoroquinolones and sulfamethoxazole-trimethoprim have decreased but are still high and further research is needed to follow prescribing patterns and enhance more prudent prescribing to this group of patients. It is important to follow changes in antibiotic treatment of infections frequently managed in primary care in order to set treatment goals and monitor adherence to clinical guidelines so as to be able to improve antibiotic management.

## Supporting information

S1 Appendix Diagnostic codes. List of diagnostic codes for lower urinary tract infection and pyelonephritis.
(DOCX)

S2 Appendix Sensitivity analysis. Characteristics of the infectious disease dataset and the urinary tract infection dataset.
(DOCX)
