# Supplementary material for: Increased adherence to treatment guidelines in patients with urinary tract infection in primary care: A retrospective study
Source: PLoS One. 2019 Mar 28;14(3):e0214572. doi: 10.1371/journal.pone.0214572 (PMC6438509; doi:10.1371/journal.pone.0214572)
Supplement: S1 Appendix — List of diagnostic codes for lower urinary tract infection and pyelonephritis. (DOCX) [file pone.0214572.s001.docx]

**S1 Appendix. Diagnostic codes.**

List of diagnostic codes for lower urinary tract infection (LUTI) and pyelonephritis ICD-10 code system or the primary care version, KSH 97P.

**List of diagnostic codes for lower Urinary tract infection**

| N30P | LUTI |
| --- | --- |
| N309 | Cystitis/ LUTI |
| N390X | LUTI |
| N390 | LUTI |
| N300 | Acute cystitis/LUTI |
| N30 | LUTI |
| N301 | LUTI |
| N302 | LUTI |
| N308 | LUTI |
| O862 | LUTI after delivery |

**List of diagnostic codes for pyelonephritis**

| N12 | pyelonephritis |
| --- | --- |
| N12P | Pyelonephritis |
| N129 | Pyelonephritis |
| N109 | Pyelonephritis |
| N110 | Pyelonephritis |
| N111 | Pyelonephritis |
| N118 | Pyelonephritis |
| N119 | pyelonephritis |
