# Supplementary material for: Increased adherence to treatment guidelines in patients with urinary tract infection in primary care: A retrospective study
Source: PLoS One. 2019 Mar 28;14(3):e0214572. doi: 10.1371/journal.pone.0214572 (PMC6438509; doi:10.1371/journal.pone.0214572)
Supplement: S2 Appendix — Characteristics of the infectious disease dataset and the urinary tract infection dataset. (DOCX) [file pone.0214572.s002.docx]

# S 2 Appendix. Sensitivity analysis.

## Characteristics of the infectious disease dataset.

|  | 2008 | | 2013 | |
| --- | --- | --- | --- | --- |
|  |  |  |  |  |
|  | All | Constant 37 | All | Constant 37 |
| Number of Primary Healthcare Centres, *n* (PHCC) | 47 | 37 | 88 | 37 |
| Number of registered persons, *n* (population) | 460 529 | 369 548 | 785 070 | 379 338 |
| Number of consultations (all causes), *n* | 662 184 | 513 698 | 1 085 829 | 563 107 |
| Consultations due to infections, *n* (percentage of all consultations %) | 210 388 (31.8) | 162 890 (31.7) | 318 976 (29.4) | 153 193 (27.2) |
| Prescribing proportions (%) | 53.7 | 51.8 | 38.6 | 38.9 |
| Prescriptions per 1000 registered persons and year | 245 | 228 | 157 | 157 |

Consultation rates and antibiotic prescribing rates were calculated for all participating PHCCs and for the constant 37 PHCCs, participating all three years. Reductions in antibiotic prescribing rate for all PHCCs and for the constant 37 PHCCs were significant, p<0.01 (*p*-values calculated using chi- 2 test).

## Characteristics of the urinary tract infection dataset.

|  | 2008 | | 2013 | |
| --- | --- | --- | --- | --- |
|  |  |  |  |  |
|  | All | Constant 37 | All | Constant 37 |
| Number of Primary Healthcare Centres, *n* (PHCC) | 47 | 37 | 88 | 37 |
| Number of consultations due to LUTI, *n* | 18 312 | 13 483 | 33 308 | 15 796 |
| Number of consultations due to LUTI women, *n* | 16 189 | 11 980 | 28 642 | 13 672 |
| Number of consultations due to LUTI men, *n* | 2123 | 1503 | 4666 | 2120 |
| Number of consultations due to pyelonephritis (men and women), *n* | 594 | 452 | 1043 | 476 |
| Consultations due to UTI (percentage of all consultations) % | 2.8 | 2.6 | 3.1 | 2.8 |
| Percentage first line^1^ antibiotic, LUTI in women, % | 58.2 | 59.3 | 69.4 | 68.5 |
| Percentage second line^2^ antibiotic, LUTI in women, % | 24.1 | 23.1 | 7.3 | 7.7 |

Consultation rates and antibiotic prescribing rates were calculated for all participating PHCCs and for the constant 37 PHCCs, participating all three years.

1. First line antibiotic: pivmecillinam, nitrofurantoin
2. Second line antibiotic: Trimethoprim, fluoroquinolones
